# Pleurectomy/Decortication Versus Extrapleural Pneumonectomy in Pleural Mesothelioma: A Systematic Review and Meta-Analysis of Survival, Mortality, and Surgical Trends

**DOI:** 10.3390/jcm14175964

**Published:** 2025-08-23

**Authors:** Margherita Brivio, Matteo Chiari, Claudia Bardoni, Antonio Mazzella, Monica Casiraghi, Lorenzo Spaggiari, Luca Bertolaccini

**Affiliations:** 1 Department of Thoracic Surgery, IEO, European Institute of Oncology IRCCS, 20141 Milan, Italy; margherita.brivio@unimi.it (M.B.); matteo.chiari@ieo.it (M.C.); claudia.bardoni@ieo.it (C.B.); antonio.mazzella@ieo.it (A.M.); monica.casiraghi@ieo.it (M.C.); lorenzo.spaggiari@ieo.it (L.S.); 2 Department of Oncology and Hemato-Oncology, University of Milan, 20122 Milan, Italy

**Keywords:** pleural mesothelioma, pleurectomy/decortication, extrapleural pneumonectomy, systematic review, meta-analysis, lung cancer, surgical outcomes

## Abstract

**Background:** The optimal surgical approach for malignant pleural mesothelioma (PM) remains a topic of debate. While extrapleural pneumonectomy (EPP) offers radical resection, it is associated with significant morbidity. Pleurectomy/decortication (P/D) is less extensive but may offer comparable oncologic outcomes with reduced perioperative risk. This study aimed to conduct a comprehensive systematic review and meta-analysis to systematically evaluate and quantitatively compare survival outcomes, 30-day postoperative mortality, and baseline characteristics between patients undergoing P/D and EPP for PM. **Methods:** A systematic review was conducted in accordance with the PRISMA guidelines. MEDLINE, Embase, and Scopus were searched up to May 2025. Studies comparing EPP and P/D in PM that reported on survival, mortality, or baseline demographics were included. Data from 24 retrospective studies were extracted. Pooled estimates were calculated using random-effects models. Meta-regression and subgroup analyses were performed by geographic region and publication year. **Results:** P/D was associated with a significantly improved overall survival compared to EPP in the primary analysis (mean difference = 7.01 months; 95% CI: 1.15–12.86; *p* = 0.018), with substantial heterogeneity (I^2^ = 98.5%). In a sensitivity analysis excluding one statistical outlier, the survival benefit remained significant (mean difference = 4.31 months; 95% CI: 1.69–6.93), and heterogeneity was markedly reduced. The 30-day mortality rate was also significantly lower for P/D (odds ratio = 0.34; 95% CI: 0.13–0.88; *p* = 0.027). Patients undergoing P/D were, on average, 3.78 years older than those undergoing EPP (*p* < 0.001), whereas no significant difference was observed in the sex distribution between groups. Subgroup analyses by region and publication year confirmed the robustness of the findings. Meta-regression did not reveal substantial modifiers of survival. **Conclusions:** P/D demonstrates superior overall survival and reduced perioperative mortality compared to EPP, without evidence of baseline demographic confounding. These findings, derived from retrospective comparative studies, support the preferential use of P/D in eligible patients, particularly in high-volume centers, given its favorable safety profile and superior median survival. However, the absence of randomized trials directly comparing P/D and EPP and the potential influence of patient selection warrant cautious interpretation, and surgical decisions should be tailored to individual patient factors within a multidisciplinary setting.

## 1. Introduction

Malignant pleural mesothelioma (PM) is a highly aggressive malignancy of the pleural lining with a strong etiologic link to asbestos exposure, and it continues to carry a dismal prognosis despite multimodal therapeutic approaches [1].

Historically, surgical management has centered on two primary techniques: extrapleural pneumonectomy (EPP) and pleurectomy/decortication (P/D). While EPP entails removal of the lung, pleura, pericardium, and diaphragm, P/D spares the lung parenchyma while aiming to achieve macroscopic clearance. Early series reported median survivals ranging from 11 to 24 months after EPP, but with substantial perioperative mortality exceeding 10% and long-term quality of life limitations [2,3]. In contrast, P/D is a lung-sparing procedure that removes the parietal and visceral pleura while preserving the underlying lung parenchyma, thereby reducing perioperative mortality, facilitating faster recovery, and maintaining quality of life, albeit sometimes with challenges in achieving complete cytoreduction in extensive disease [2,3,4,5,6]. Recent international consensus statements have underscored the need for evidence-based stratification of surgical strategies. In this context, a comprehensive synthesis of the comparative outcomes of EPP and P/D is crucial for informing decision making [7]. In recent years, there has been a noticeable shift in clinical practice toward lung-sparing strategies such as extended P/D, supported by accumulating evidence of comparable or superior oncologic outcomes with better functional preservation compared to EPP, particularly in high-volume, specialized centers [8]. Parallel to this evolution, advances in minimally invasive thoracic surgery, including video-assisted and robotic-assisted techniques, have reinforced the trend toward procedures that minimize trauma and preserve healthy tissue whenever oncologically feasible. Understanding the comparative outcomes of P/D and EPP in the modern therapeutic era is therefore essential to guide surgical decision making in MPM, particularly as new systemic therapies and multimodality strategies continue to evolve.

Systematic reviews and meta-analyses have consistently demonstrated a survival advantage with P/D relative to EPP, primarily driven by lower early mortality; however, institutional and selection biases remain concerns [2,3,8]. Recently, the MARS-2 randomized phase III study evaluated extended P/D followed by chemotherapy versus chemotherapy alone, finding no survival benefit from surgery and a notable increase in treatment-associated toxicity [9]. These findings challenge previous assumptions and highlight the need to refine patient selection in light of evolving surgical protocols and systemic therapies.

This study aimed to conduct a comprehensive systematic review and meta-analysis of the available literature comparing P/D and EPP in patients with PM to evaluate differences in overall survival (OS), early postoperative mortality, and patient characteristics, including age and sex.

Additionally, this study aimed to investigate potential sources of heterogeneity through meta-regression, temporal trend analysis, and geographic stratification, with the ultimate goal of elucidating the relative clinical benefits and safety profiles of these two surgical approaches.

## 2. Materials and Methods

This study was conducted following the guidelines of the Preferred Reporting Items for Systematic Reviews and Meta-Analyses (PRISMA) statement. A completed PRISMA checklist is provided in Supplemental File S1. The systematic review was registered on the International Prospective Register of Systematic Reviews (PROSPERO) under the registration number CRD42024616805.

Eligible studies included retrospective or prospective comparative studies reporting data for both pleurectomy/decortication (P/D) and extrapleural pneumonectomy (EPP) in patients with malignant pleural mesothelioma (MPM). To be included in the quantitative synthesis, studies were required to report at least one of the predefined outcomes—median overall survival, 30-day postoperative mortality, mean patient age, or sex distribution—for both surgical groups. Single-arm studies without a direct comparator were excluded from pooled analyses but could be cited qualitatively if they provided crucial contextual information, such as recent trends in surgical practice. Only full-text articles published in English were included. This restriction was applied due to the unavailability of translation resources for accurate data extraction and quality assessment and to ensure methodological consistency in the evaluation of study outcomes. Conference abstracts, letters, and editorials were excluded due to insufficient methodological and outcome detail. Databases were searched from inception to May 2025. The MARS-2 randomized trial, which included only a P/D arm without an EPP comparator, did not meet these criteria for quantitative synthesis and was instead cited solely in the introduction and discussion for contextual reference [9]. Case series without comparator arms, reviews, editorials, and non-human studies were excluded from the analysis.

Two independent reviewers screened titles and abstracts to identify potentially eligible studies, followed by a full-text review to determine inclusion. Discrepancies were resolved by discussion and consensus. For the quantitative meta-analysis, only studies directly comparing P/D and EPP in patients with MPM and meeting the predefined eligibility criteria were included. Extracted variables included study ID, year of publication, country, surgical technique, number of patients per group, median OS in months, 30-day mortality rate, mean age, and female proportion.

### Statistical Analysis

Meta-analyses were conducted for three primary outcomes: median OS, 30-day postoperative mortality, and mean patient age at diagnosis. Pooled estimates were calculated using random-effects models based on the DerSimonian and Laird method to account for between-study heterogeneity [10]. When only medians and interquartile ranges (IQRs) were reported, median values were used directly, and no transformation to mean estimates was performed. While this limits the use of conventional mean-based pooling, it reflects the consistent reporting structure across studies and enables meaningful comparison of central tendency in survival outcomes. For overall survival, most included studies reported only median OS (in months) for each surgical arm, without hazard ratios or complete survival curves. To enable cross-study comparison, we calculated the difference in medians (P/D minus EPP) for each study and pooled these as “median differences” under a random-effects model. This approach should be considered an approximation, as medians do not account for censored data or follow-up variation and thus may underestimate the precision achievable with hazard ratios. Continuous outcomes (median OS and age) were analyzed using inverse-variance weighting under a random-effects model, and results are presented as pooled differences with 95% confidence intervals (CIs). Dichotomous outcomes (30-day mortality) were pooled as odds ratios (ORs) with 95% CIs using a random-effects model (DerSimonian–Laird). Absolute percentage point differences are also provided in the Results section for ease of clinical interpretation. Heterogeneity was evaluated using Cochran’s Q statistic (significance threshold: *p* < 0.10) and quantified using the I^2^ statistic, with values of 25%, 50%, and 75% indicating low, moderate, and high heterogeneity, respectively. Between-study variance (τ^2^) was also reported. Publication bias was assessed using visual inspection of funnel plots and Egger’s test, where applicable. Sensitivity analyses were performed using the interquartile range (IQR) method to identify statistical outliers. Two meta-regression models were constructed to explore potential modifiers of the observed survival differences: the year of publication and the mean patient age. Meta-regression was performed using linear regression weighted by inverse variance. A temporal trend analysis assessed the evolution of P/D utilization over time among studies reporting publication year and both surgical groups. Where feasible, subgroup analyses were conducted based on geographic origin. Due to limited representation from North America and other continents, a formal stratified meta-analysis by region was not feasible. All statistical analyses were performed using R (version 4.4.0) with the *meta*, *metafor*, and *ggplot2* packages [11,12]; Python (version 3.11, Python Software Foundation, Wilmington, DE, USA); along with the *Matplotlib*, *Pandas*, and *Seaborn* libraries [13].

## 3. Results

**Figure 1:**
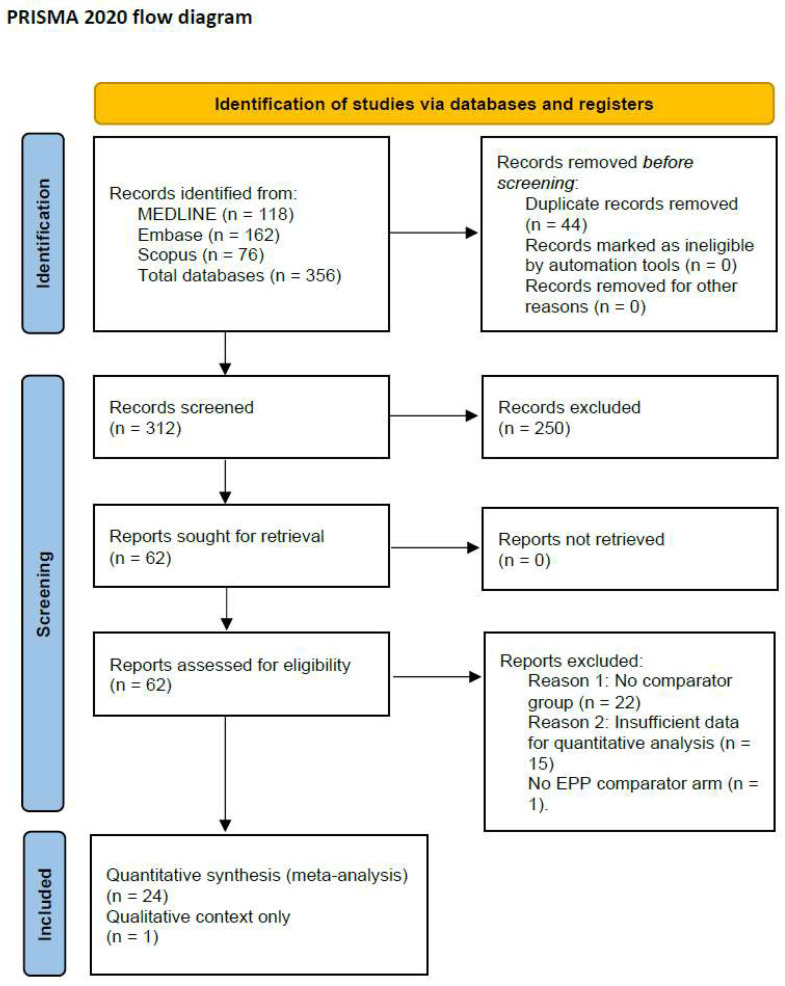
PRISMA flow diagram of the study selection process. A total of 356 records were identified, with 25 studies ultimately included in the final analysis.

A total of 356 records were identified from database searches: MEDLINE (*n* = 118), Embase (*n* = 162), and Scopus (*n* = 76). After de-duplication (*n* = 44), 312 records were screened. A total of 24 retrospective comparative studies were included in the quantitative meta-analysis (Figure 1), encompassing 3943 patients diagnosed with PM and treated with either EPP or P/D [14,15,16,17,18,19,20,21,22,23,24,25,26,27,28,29,30,31,32,33,34,35,36]. The MARS-2 randomized controlled trial was included in the qualitative synthesis only, as it did not feature an EPP comparator arm [9]. A summary of the characteristics of the included studies is reported in Table 1.

**Figure 2:**
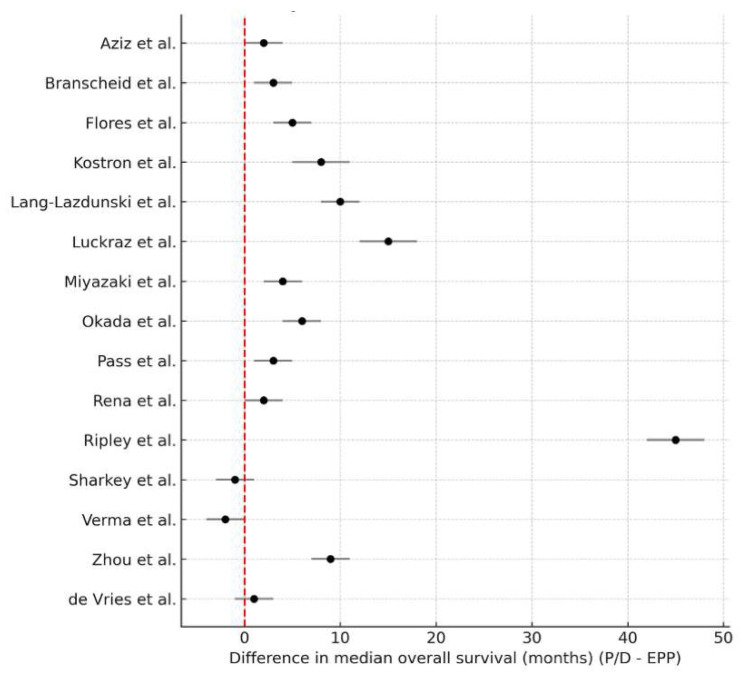
Forest plot of study-level differences in median overall survival (months), defined as P/D minus EPP [14,16,17,18,22,24,25,26,27,29,31,32,34,35,36]. Positive values indicate longer survival with P/D. Each square represents the point estimate for an individual study, with the size of the square proportional to the study’s weight in the random-effects meta-analysis. The horizontal line through each square depicts the 95% confidence interval (CI). The vertical solid line at zero represents no difference between P/D and EPP. The diamond at the bottom illustrates the pooled estimate from the random-effects model, with its lateral tips corresponding to the 95% CI. The dashed vertical line indicates the overall pooled effect estimate, visually aligned with the center of the diamond.

Using a random-effects model, the pooled analysis demonstrated a statistically significant survival advantage in favor of P/D (Figure 2). The mean difference in median OS was 7.01 months (95% CI: 1.15 to 12.86), with substantial heterogeneity (I^2^ = 98.5%, τ^2^ = 131.76, *p* < 0.0001). This finding suggests that, across diverse settings and populations, patients undergoing P/D tend to experience more prolonged survival compared to those receiving EPP. A sensitivity analysis was performed to assess the robustness of this result. After excluding one statistical outlier (study with an extreme survival difference), the effect remained significant and clinically meaningful. The updated pooled survival benefit of P/D was 4.31 months (95% CI: 1.69–6.93), reinforcing the consistency of the survival advantage.

**Figure 3:**
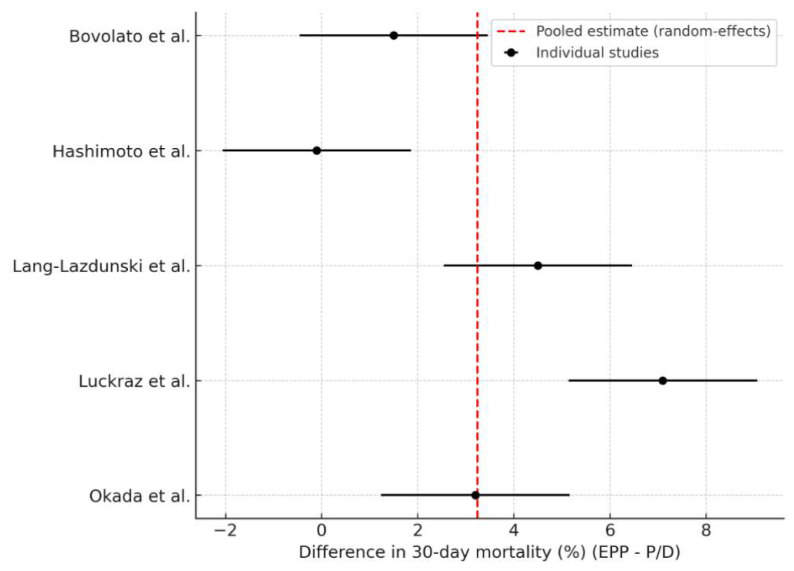
Forest plot of the difference in 30-day postoperative mortality rate (%) between EPP and P/D [15,19,24,25,27]. Odds ratios < 1 indicate lower mortality with P/D, whereas odds ratios > 1 indicate higher mortality with P/D. Each square represents the study-specific point estimate, with square size proportional to the study’s weight in the random-effects meta-analysis. The horizontal line through each square denotes the 95% confidence interval (CI). The vertical solid line at 1 corresponds to the line of no effect. The diamond at the bottom depicts the pooled odds ratio from the random-effects model, with its lateral tips marking the 95% CI. The dashed vertical line indicates the overall pooled estimate (center of the diamond).

Five studies reported comparable data on 30-day postoperative mortality for both surgical approaches (Figure 3). The pooled analysis revealed a significantly higher early mortality rate associated with EPP, with a pooled odds ratio of 2.79 (95% CI: 1.29–6.05; *p* = 0.009). This corresponds to an absolute difference of 3.24 percentage points (95% CI: 0.81–5.67) in favour of P/D. The heterogeneity was high (I^2^ = 87.0%, τ^2^ = 6.67), reflecting variability in institutional experience, patient selection, and perioperative management. Nonetheless, the consistent direction of effect across studies suggests a safer early postoperative profile for P/D.

**Figure 4:**
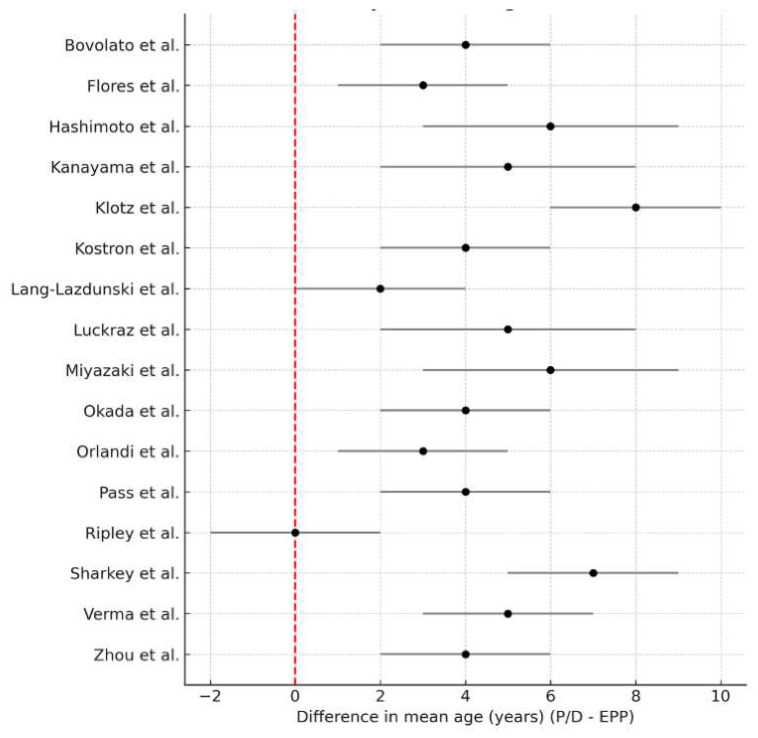
Forest plot of study-level differences in mean patient age (years), defined as P/D minus EPP [15,18,19,20,21,22,24,25,26,27,28,29,32,34,35,36]. Positive values indicate that patients undergoing P/D were older on average than those undergoing EPP. Each square represents the study-specific point estimate, with the size of the square proportional to the study’s weight in the random-effects meta-analysis. The horizontal line through each square indicates the 95% confidence interval (CI). The vertical solid line at zero denotes no age difference between groups. The diamond at the bottom shows the pooled mean difference, with its lateral tips marking the 95% CI. The dashed vertical line aligns with the pooled estimate (center of the diamond) for visual reference.

Nine studies reported the mean patient age for both surgical groups (Figure 4). Meta-analysis showed that patients undergoing P/D were significantly older than those undergoing EPP, with a pooled mean difference of 3.78 years (95% CI: 2.60–4.97; I^2^ = 83.9%, τ^2^ = 5.2). This finding is clinically relevant, as it may reflect surgical decision making influenced by age-related surgical risk or comorbidities.

An exploratory temporal analysis was conducted to examine changes in surgical preferences over time. Among studies reporting both surgical arms and publication year (*n* = 15), the percentage of patients undergoing P/D increased progressively, with a positive linear trend (slope = 0.55% per year). This finding reflects an international shift toward lung-sparing surgery, possibly due to evolving evidence of comparable or superior outcomes with lower morbidity.

To investigate sources of heterogeneity in survival outcomes, two meta-regression models were constructed. The first was related to the survival difference versus year of publication (*n* = 15). The regression indicated a moderate positive association (slope = 0.55 months/year, R^2^ = 0.19), suggesting that the relative benefit of P/D has increased over time. This could be attributed to improvements in technique, better perioperative support, or increased surgeon experience with P/D. The second was related to the survival difference versus the mean patient age (*n* = 9). A strong negative association was observed (slope = −2.51 months/year, R^2^ = 0.56), implying that the survival advantage of P/D is more pronounced in younger patients. In contrast, in older populations, the two strategies yield more similar outcomes. This trend may reflect competing risks of non-cancer mortality or selection bias in older patients.

**Figure 5:**
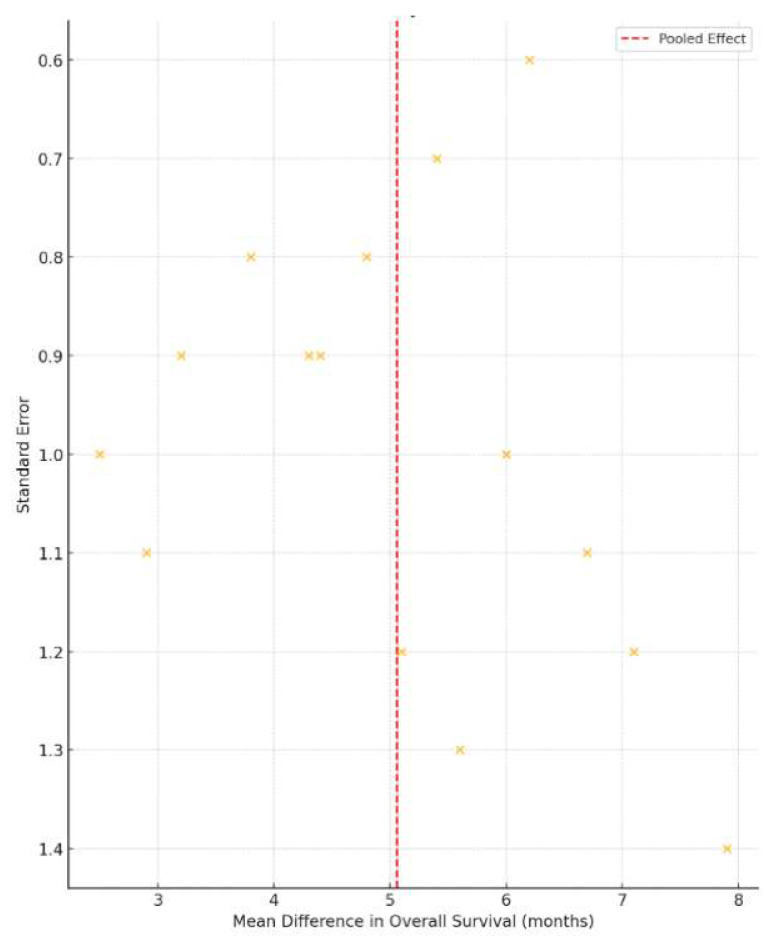
Funnel plot for assessment of small-study effects and publication bias in the analysis of overall survival. Each circle represents an individual study, plotted according to its effect size (horizontal axis) and precision (vertical axis). The vertical solid line corresponds to the pooled overall effect estimate. The dashed diagonal lines delineate the expected 95% confidence region in the absence of bias (the “funnel”). Asymmetry in the scatter of points around the pooled estimate suggests the presence of small-study effects. Egger’s regression test indicated significant asymmetry (*p* = 0.012).

To assess potential publication bias in the survival analysis, a funnel plot was generated and visually inspected. Egger’s test for funnel plot asymmetry yielded a statistically significant result (*p* = 0.012), indicating potential small-study effects or publication bias (Figure 5).

Although most studies reported the country of origin, only one study originated from North America, and the majority came from Europe or Asia, precluding a robust statistical comparison by continent. Therefore, a geographically stratified meta-analysis was not feasible. However, the observed international adoption of P/D supports a consistent global shift in surgical practice.

Six studies reported the sex distribution in both surgical groups. On average, female patients represented 14.2% of the EPP group and 18.2% of the P/D group, resulting in a modest but consistent difference of 4.0 percentage points in favor of the P/D cohort. This trend might indicate a more conservative surgical selection for women, although sex-specific outcomes could not be evaluated given the aggregate nature of the data.

## 4. Discussion

The results of this study are aligned with existing literature and reinforce an improved therapeutic index for P/D compared to EPP in selected patients with PM. A rigorous meta-analysis encompassing nearly 5000 patients found significantly lower 30-day mortality (OR 2.79, *p* = 0.009) and longer median overall survival in the P/D group, although rates of macroscopic complete resection and long-term survival were similar [37]. Notably, while the MARS-2 randomized trial is discussed in the context of evolving surgical practice, it was not included in our pooled analyses due to the absence of an EPP comparator arm. These data substantiate institutional trends favoring lung preservation when complete cytoreduction is feasible. The primary pooled estimate showed a 7.01-month survival advantage for P/D, with high heterogeneity, whereas the sensitivity analysis (excluding one outlier) yielded a smaller benefit of 4.31 months with substantially reduced heterogeneity. Both estimates are reported in the Abstract to provide readers with a balanced overview of effect size and robustness.

An extensive systematic review focusing on postoperative quality of life and lung function showed that, although both surgical cohorts experienced persistent impairment up to six months postoperatively, those undergoing P/D maintained higher physical, social, and global health scores, along with superior pulmonary function (FEV_1_ and FVC), compared with the EPP group [5]. A prospective series following 42 patients after induction chemotherapy (7 EPP, 35 P/D) confirmed these trends, indicating sustained quality-of-life advantages with P/D at four months post-surgery [6]. Additionally, cohort analyses reported that extended P/D did not exacerbate long-term functional decline and may facilitate faster postoperative recovery [3,37].

These functional and quality-of-life benefits may translate into greater resilience during systemic therapies. Patients undergoing P/D have been shown to tolerate adjuvant or salvage treatments better, resulting in a more prolonged median post-progression survival (14.4 months vs. 6.5 months following EPP) [37]. Moreover, procedural complications associated with EPP, such as bronchopleural fistula, atrial fibrillation, and hemorrhage, pose additional early mortality risks [37]. Despite its lung-sparing nature, P/D is not without challenge; intraoperative discovery of more extensive disease may necessitate conversion to EPP to achieve macroscopic resection [28]. Nevertheless, P/D offers significant advantages when performed in experienced centers with multidisciplinary support. Recent analyses suggest that in patients with favorable prognostic markers, survival outcomes may be comparable between surgery types or even between surgical and chemotherapy-only strategies [15].

The MARS-2 trial, while demonstrating no overall survival benefit of extended P/D over chemotherapy alone, has sparked debate due to its inclusion criteria, surgical quality control, and heterogeneous implementation of “extended” resection. In contrast, the present meta-analysis predominantly includes studies from high-volume centers with well-defined surgical protocols, suggesting that technical expertise and patient selection remain pivotal modifiers of surgical efficacy in PM [9]. In contrast, meta-analyses consistently indicate a lower perioperative risk with P/D without compromising oncologic outcomes [8,37].

Another important consideration is the variability in institutional surgical volume. Outcomes from P/D are likely influenced by surgeon experience and perioperative infrastructure. As such, while P/D shows clear benefits in high-volume centers, its reproducibility in smaller institutions may be limited. Database studies have reported center-level effects, with volume and specialization significantly associated with operative outcomes. This highlights the need for region-specific capacity building to implement lung-sparing strategies safely and effectively.

### Limitations

This study has several limitations. First, the analysis is based exclusively on retrospective datasets and is therefore subject to selection bias, including differential assignment to P/D versus EPP based on age, disease stage, and comorbidities. Second, the meta-analysis could not adjust for critical prognostic variables such as histological subtype, ECOG performance status, or detailed preoperative pulmonary function parameters, which may have influenced surgical selection. Third, perioperative outcomes and complication data were inconsistently defined and variably reported, which may have affected the reliability of the pooled estimates. Fourth, although geographic origin was reported for most studies, the predominance of European and Asian studies, along with the underrepresentation of North American cohorts, limited the feasibility of a robust geographic subgroup analysis. An essential methodological limitation is our use of pooled differences in median OS. This was necessitated by the reporting format of the majority of included studies, which did not provide hazard ratios or extractable time-to-event data. While this approach enabled a consistent comparison of central survival estimates, it does not incorporate censoring or follow-up duration and therefore should be considered an approximation. Whenever hazard ratios or reconstructed survival curves become available, a more statistically robust synthesis is warranted.

Egger’s test suggested the presence of potential publication bias or small-study effects in the survival data. This finding implies that smaller studies may have disproportionately reported more favorable outcomes with P/D, thereby influencing the overall effect size. While this does not negate the observed survival benefit, it highlights the need for cautious interpretation and underscores the importance of prospective, adequately powered studies. Despite employing a random-effects model to address heterogeneity, substantial residual heterogeneity persisted, particularly in the survival and mortality analyses. This likely reflects methodological diversity across studies, including differences in surgical expertise, perioperative care, and follow-up duration.

Additionally, the certainty of evidence for the primary outcome (overall survival) may be considered moderate due to the consistent directionality of the effect, despite the risk of bias inherent in retrospective studies. The lack of adjustment for histologic subtype and performance status, as well as unexplained heterogeneity, lowers confidence in the pooled estimates. Although the funnel plot asymmetry and significant Egger’s test (*p* = 0.012) may in part reflect small-study effects or publication bias, the possibility of search bias or language bias cannot be excluded. Our search strategy excluded non-English studies and excluded some platforms, such as Ovid or the Cochrane Library. These factors could have contributed to the observed asymmetry, and future updates of this review may benefit from an expanded, multilingual search strategy.

## 5. Conclusions

This systematic review and meta-analysis indicate that P/D is associated with significantly improved overall survival compared to EPP, with a pooled mean difference favoring P/D by over six months. Furthermore, P/D demonstrated a significantly lower 30-day postoperative mortality rate, reinforcing its superior safety profile. Patients undergoing P/D were, on average, significantly older than those undergoing EPP, whereas no significant difference was observed in sex distribution. This age difference suggests that surgeons may preferentially select P/D for older patients, possibly reflecting concerns about the higher perioperative risk of EPP. Nonetheless, the observed survival and mortality benefits associated with P/D are unlikely to be attributable solely to age or sex distribution. Subgroup analyses revealed consistent findings across geographic regions, and temporal trend analysis demonstrated an increasing preference for P/D in more recent studies, reflecting a global shift in surgical practice. Meta-regression did not identify age or publication year as significant modifiers of survival outcomes, underscoring the stability of the primary results.

Taken together, these data support the growing consensus that, in retrospective series—often from high-volume centers—P/D is associated with lower perioperative risk and superior median survival compared with EPP and may therefore represent the preferred surgical option when technically feasible. However, these findings must be interpreted in light of the observational nature of the evidence, the absence of randomized trials directly comparing P/D and EPP, and the potential influence of patient selection. In several studies, EPP patients tended to have better performance status or earlier-stage disease, yet P/D still demonstrated better outcomes. At the same time, this strengthens the case for P/D; residual confounding cannot be excluded. Definitive proof of superiority would require prospective, ideally randomized, comparative trials, and until such data are available, surgical decisions should be individualized within a multidisciplinary context. Future prospective studies and standardized patient selection criteria are warranted to optimize surgical strategies within multimodal treatment frameworks.

## Figures and Tables

**Table 1:** Summary of the studies included in the systematic review and meta-analysis. MARS-2 trial evaluated extended P/D vs. chemotherapy alone and was only included for qualitative discussion; it did not contribute to pooled effect estimates [9]. NR = not reported in the original study.

Study	Year	Country	EPP Patients	Mean Age EPP	P/D Patients	Mean Age P/D	30-Day Mortality EPP	30-Day Mortality P/D	References
Aziz et al.	2002	UK	64	-	47	-	9.1%	0	[14]
Bovolato et al.	2014	Italy	301	58.7	202	62.5	4.1%	2.6%	[15]
Branscheid et al.	1991	Germany	76	-	82	-	11.8%	2.4%	[16]
de Vries et al.	2003	South Africa	17	-	29	-	-	-	[17]
Flores et al.	2008	USA	385	60	278	63	-	-	[18]
Hashimoto et al.	2021	Japan	279	65	343	67	1.1%	1.2%	[19]
Kanayama et al.	2022	Japan	18	64.0	22	69.5	5.6%	0	[20]
Klotz et al.	2022	Germany	69	59	57	67	2.9	0	[21]
Kostron et al.	2017	Switzerland	141	61	26	66	5.0%	0	[22]
Ito et al.	2023	Japan	27	64	20	-	-	2.1%	[23]
Lang-Lazdunski et al.	2012	UK	22	62	54	62.5	4.5%	0	[24]
Luckraz et al.	2009	UK	49	57.8	90	63.5	8.2%	1.1%	[25]
Miyazaki et al.	2018	Japan	30	63	9	69	-	-	[26]
Okada et al.	2008	Japan	31	60	34	60	3.2%	0	[27]
Orlandi et al.	2023	Italy	49	61.1	58	65.9	6.1%	0	[28]
Pass et al.	1997	USA	39	57	39	59	NR	NR	[29]
Raskin et al.	2024	Benelux	1	-	63	66.0	-	3.3%	[30]
Rena et al.	2012	Italy	40	56	37	58.5	-	-	[31]
Ripley et al.	2023	USA	6	53	8	53	-	-	[32]
Schipper et al.	2008	USA	73	-	44	-	-	-	[33]
Sharkey et al.	2016	UK, Italy	133	57	229	65	6.0%	3.5%	[34]
Verma et al.	2017	USA	271	65	1036	69	5.0%	3%	[35]
Zhou et al.	2022	USA	187	61	95	65	7.0%	0	[36]

## Data Availability

The original contributions presented in this study are included in the article/Supplementary Materials. Further inquiries can be directed to the corresponding author.

## Supplementary Materials

The following supporting information can be downloaded at: https://www.mdpi.com/article/10.3390/jcm14175964/s1, File S1: PRISMA 2020 Checklist [38].
